# Chemotaxonomic Characterization and *in-Vitro* Antimicrobial and Cytotoxic Activities of the Leaf Essential Oil of *Curcuma longa* Grown in Southern Nigeria

**DOI:** 10.3390/medicines2040340

**Published:** 2015-12-21

**Authors:** Emmanuel E. Essien, Jennifer Schmidt Newby, Tameka M. Walker, William N. Setzer, Olusegun Ekundayo

**Affiliations:** 1 Department of Chemistry, University of Uyo, Akwa Ibom State 520101, Nigeria; 2 Department of Chemistry, University of Alabama in Huntsville, Huntsville, AL 35899, USA; E-Mails: jennifer.newby315@gmail.com (J.S.N.); Ivygrad08@yahoo.com (T.M.W.); wsetzer@chemistry.uah.edu (W.N.S.); 3 Department of Chemistry, University of Ibadan, Ibadan 200284, Nigeria; E-Mail: oekundayo@yahoo.com

**Keywords:** *Curcuma longa*, essential oil composition, *ar*-turmerone, α-turmerone, β-turmerone, antimicrobial, cytotoxic

## Abstract

*Curcuma longa* (turmeric) has been used in Chinese traditional medicine and Ayurvedic medicine for many years. Methods: The leaf essential oil of *C. longa* from southern Nigeria was obtained by hydrodistillation and analyzed by gas chromatography–mass spectrometry (GC-MS). The essential oil was screened for *in vitro* antibacterial, antifungal, and cytotoxic activities. The major components in *C. longa* leaf oil were *ar*-turmerone (63.4%), α-turmerone (13.7%), and β-turmerone (12.6%). A cluster analysis has revealed this to be a new essential oil chemotype of *C. longa*. The leaf oil showed notable antibacterial activity to *Bacillus cereus* and *Staphylococcus aureus*, antifungal activity to *Aspergillus niger*, and cytotoxic activity to Hs 578T (breast tumor) and PC-3 (prostate tumor) cells. The *ar*-turmerone-rich leaf essential oil of *C. longa* from Nigeria has shown potent biological activity and therapeutic promise.

## 1. Introduction

*Curcuma longa* L. (syn. *C. domestica* Vahl.) is a perennial rhizomatous herb of the family Zingiberaceae. The rhizome is the source of turmeric, which has use as a condiment and coloring agent in medicines, confectionery and curry powder [1]. Turmeric has a long history of traditional use in the Chinese and Ayurvedic systems of medicine, particularly as an anti-inflammatory agent [2], and for the treatment of biliary disorders, anorexia, coryza, cough, diabetic wounds, hepatic disorders, rheumatism and sinusitis [1]. The powdered rhizome is used externally as an antiseptic [3] and taken internally to cure gastritis [4]. The rhizome volatiles have been extensively studied (see, for example [5,6,7,8]), and there have been previous reports on the leaf essential oil compositions [9,10,11,12,13,14,15,16,17,18,19,20,21,22,23,24,25,26]. Research has shown that the quantitative essential oil composition is widely influenced by the genotype, ontogenic development, and environmental and growing conditions [27,28,29]. It also implies the possibility of different medicinal uses of the same plant species grown in different regions [30]. This paper reports the chemical constitution of the leaf essential oil of *C. longa* grown in the southern part of Nigeria and its antimicrobial effects and anti-neoplastic potential.

## 2. Experimental Section

### 2.1. Plant Material

Fully grown leaves of *C. longa* were collected from plants cultivated in the village of Mbaiso, Ikot Ekpene Local Government Area of Akwa Ibom State, Nigeria, in the month of October. Plant materials were authenticated by F. Usang of the Forest Research Institute of Nigeria (FRIN), Ibadan, where voucher specimens were deposited under FHI 106920. The essential oil was obtained by hydrodistillation (4 h) of the air-dried plant leaves using a Clevenger-type apparatus in accordance with the British Pharmacopoeia [31]. The leaf oil was dried over sodium sulfate and kept in refrigeration (4 °C) after estimation of percentage yield.

### 2.2. Gas Chromatographic–Mass Spectral Analysis

The essential oil was subjected to gas chromatography-mass spectrometry (GC-MS) analysis on an Agilent system consisting of a model 6890 gas chromatograph, a model 5973 mass selective detector (MSD), and an Agilent ChemStation data system. The GC column was a Hewlett Packard (HP-5ms) fused silica capillary with a (5% phenyl)-methyl polysiloxane stationary phase (30 m × 0.25 μm film thickness). The carrier gas was helium with a column head pressure of 7.07 psi and flow rate of 1.0 mL/min. Inlet temperature was 200 °C and MSD detector temperature was 280 °C. The GC oven temperature program was used as follows: 40 °C initial temperature held for 10 min; increased at 3 °C/min to 200 °C; increased 2 °C/min to 220 °C. The sample was dissolved in CH_2_Cl_2_, and 1 μL was injected using a splitless injection technique.

Identification of individual constituents of the essential oils was achieved based on their retention indices (determined with a reference to a homologous series of normal alkanes) and by comparison of their mass spectral fragmentation patterns (National Institute of Standards and Technology, NIST database/ChemStation data system, http://www.agilent.com/chem/nds) and with the literature [32].

### 2.3. Antibacterial Screening

*Curcuma longa* leaf oil was screened for antibacterial activity against *Bacillus cereus* (ATCC No. 14579), *Staphylococcus aureus* (ATCC No. 29213), *Pseudomonas aeruginosa* (ATCC No. 27853), and *Escherichia coli* (ATCC No. 10798). Minimum inhibitory concentrations (MICs) were determined using the microbroth dilution technique [33]. Dilutions of the samples were prepared in cation-adjusted Mueller Hinton broth (CAMHB) beginning with 50 μL of 1% *w*/*w* solutions of samples in dimethylsulfoxide (DMSO) plus 50 μL CAMHB. The sample solutions were serially diluted (1:1) in CAMHB in 96-well plates to give concentrations of 2500, 1250, 625, 313, 156, 78, 39, and 19.5 μg/mL. Organisms at a concentration of approximately 1.5 × 10^8^ colony-forming units (CFU)/mL were added to each well. Plates were incubated at 37 °C for 24 h; the final minimum inhibitory concentration (MIC) was determined as the lowest concentration without turbidity. Gentamicin was used as a positive antibiotic control; DMSO was used as a negative control.

### 2.4. Antifungal Screening

Antifungal activity was determined, as described above for bacteria, using *Candida albicans* (ATCC No.10231) in a yeast-nitrogen base growth medium with approximately 7.5 × 10^7^ CFU/mL. Amphotericin B was used as the positive control. An additional test for antifungal activity against *Aspergillus niger* (ATCC No. 16888) was determined as above using yeast mold (YM) broth inoculated with *A. niger* hyphal culture diluted to a McFarland turbidity of 1.0. Amphotericin B was the positive control.

### 2.5. Cell Culture

Human Hs578T breast ductal carcinoma cells (ATCC No. HTB-129) [34] were grown in a 3% CO_2_ environment at 37 °C in DMEM with 4500 mg glucose per liter of medium, supplemented with 10% fetal bovine serum, 10 μg bovine insulin, 100,000 units penicillin and 10.0 mg streptomycin per liter of medium, and buffered with 44 mM NaHCO_3_, pH 7.35.

Human PC-3 prostatic carcinoma cells (ATCC No. CRL-1435) [35] were grown in a 3% CO_2_ environment at 37 °C in RPMI-1640 medium with l-glutamine, supplemented with 10% fetal bovine serum, 100,000 units penicillin and 10.0 mg streptomycin per liter of medium and buffered with 15 mM Hepes and 23.6 mM NaHCO_3_, pH 7.30.

### 2.6. Cytotoxicity Screening

Hs578T cells were plated into 96-well cell culture plates at 1.0 × 10^5^ cells per well and PC-3 cells at 1.9 × 10^4^ cells per well. The volume in each well was 100 μL for both cell types. After 48 h, supernatant fluid was removed by suction and replaced with100 μL growth medium containing either 2.5 or 1.0 μL of DMSO solution of oils (1% *w*/*w* in DMSO), giving a final concentration of 250 or 100 μg/mL, respectively, for each oil. Hs578T cells were tested with final concentrations at 250 μg/mL and PC-3 at a final concentration of 100 μg/mL. Solutions were added to wells in four replicates. Medium controls and DMSO controls (25 or 10 μL DMSO/mL) were used. Tingenone (250 or 100 μg/mL) was used as a positive control [36]. After the addition of the sample, plates were incubated for 48 h at 37 °C; medium was then removed by suction, and 100 μL of fresh medium was added to each well. In order to establish percent kill rates, the Cell Titer 96^®^AQ_ueous_ Non-Radioactive Cell Proliferation assay was performed [37]. After colorimetric readings were recorded (using a Molecular Devices SpectraMAX Plus microplate reader, Sunnyvale, CA, USA, 490 nm), average absorbances, standard deviations and percent kill ratios (% kill_oil_/% kill_DMSO_) were calculated.

### 2.7. Hierarchical Cluster Analysis

**Figure 1:**
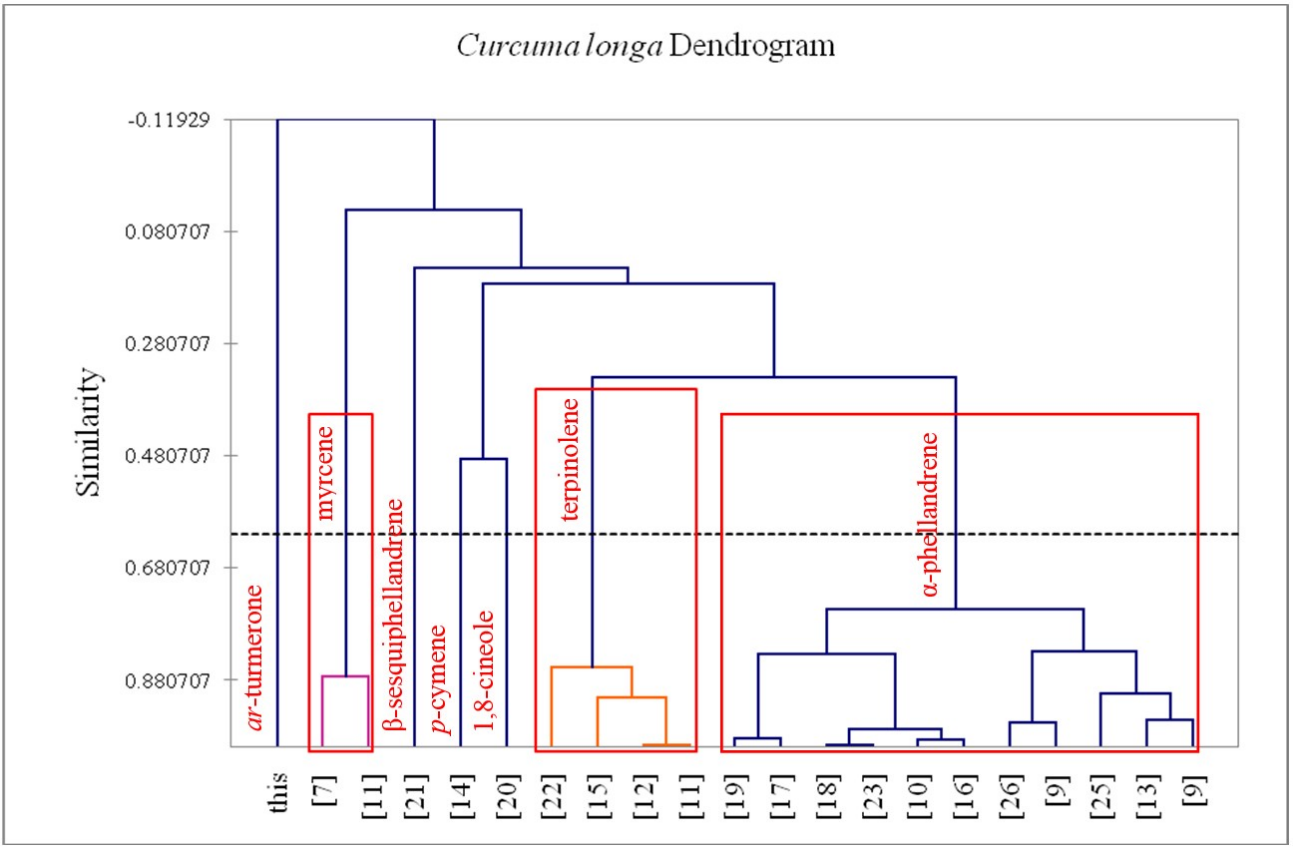
Dendrogram obtained from the agglomerative hierarchical cluster analysis of 21 *Curcuma longa* leaf essential oil samples.

A total of 20 *C. longa* leaf essential oil compositions from the published literature, as well as the composition from this study, were treated as operational taxonomic units (OTUs). The percentage composition of 21 major essential oil components (α-pinene, β-pinene, myrcene, α-phellandrene, α-terpinene, *p*-cymene, limonene, 1,8-cineole, γ-terpinene, terpinolene, linalool, terpinen-4-ol, α-terpineol, β-caryophyllene, *ar*-curcumene, α-zingiberene, β-sesquiphellandrene, *ar*-turmerone, β-turmerone, germacrone, and α-turmerone) was used to determine the chemical relationship between the various *C. longa* leaf oil samples by agglomerative hierarchical cluster (AHC) analysis using the XLSTAT software, version 2015.4.01 (Addinsoft, Brooklyn, NY, USA). Pearson correlation was selected as a measure of similarity, and the unweighted pair-group method with arithmetic average (UPGMA) was used for cluster definition. The resulting dendrogram is shown in Figure 1.

## 3. Results and Discussion

The leaf essential oil of *C. longa* was obtained in 0.67% (*w*/*w*) yield. Chemical analysis of the oil revealed 13 constituents representing 100% of the total essential oil composition (Table 1). The leaf oil was dominated by *ar*-turmerone (63.4%), with lesser quantities of α-turmerone (13.7%) and β-turmerone (12.6%). This turmerone-rich essential oil is very different from compositions of *C. longa* leaf oils published previously [7,8,9,10,11,12,13,14,15,16,17,18,19,20,21,22,23,24,25,26]. A cluster analysis of 20 published essential oil compositions in addition to the leaf oil in this present study (see Figure 1) shows seven different chemotypes: (1) an *ar*-turmerone-rich chemotype, represented by the leaf oil in this study; (2) a cluster dominated by α-phellandrene, represented by 11 samples; (3) a terpinolene-rich chemotype; (4) a β-sesquiphellandrene-rich chemotype; (5) a *p*-cymene-rich chemotype; (6) a 1,8-cineole chemotype, and (7) a myrcene chemotype. Thus, there are wide variations in *C. longa* leaf essential oils, but the oil from southern Nigeria is the only one dominated by turmerones.

**Table 1:** Chemical composition of *Curcuma longa* leaf essential oil.

RI ^a^	Compound	%	RI ^a^	Compound	%
977	β-Pinene	0.1	1524	β-Sesquiphellandrene	0.9
1023	*p*-Cymene	1.6	1633	β-Acorenol	1.0
1029	1,8-Cineole	1.6	1666	*ar*-Turmerone	63.4
1452	α-Humulene	0.2	1669	β-Turmerone	12.6
1483	*ar*-Curcumene	2.0	1705	α-Turmerone	13.7
1487	β-Ionone	0.6	1770	(*E*)-α-Atlantone	1.0
1509	β-Bisabolene	0.3	-	Total Identified	100

^a^ RI = Retention Index determined in reference to a homologous series of *n*-alkanes on an HP-5ms column.

The abundance of turmerones and the exclusive non-detection of α-phellandrene in the leaf oil sample from this work are noteworthy. α-Phellandrene has been shown to occur in high concentrations in most published data on *C. longa* leaf essential oils. There are also notable differences in the chemical profiles of leaf oils from two different regions in Nigeria. The fresh leaf essential oil of *C. longa* grown in Ile-Ife, in southwest Nigeria, where there is predominately tropical rainforest vegetation, was reported to contain α-phellandrene (47.7%) and terpinolene (28.9%) as its major constituents [19]. In the current study, the leaf was sourced from Mbaise in the southern zone of Nigeria, which is also tropical forest with comparable rainfall and temperatures. Although it is tempting to attribute the variations in chemical composition of the leaf oils to geographical location, leaf oil samples from Bhutan [9], Vietnam [13], and Malaysia [26], as well as several samples from India [9,10,16,17,18,23,25], all belong to the α-phellandrene chemotype (see Figure 1). Conversely, a sample of the “Roma” cultivar from Orissa, India, was rich in terpinolene (87.8%) [11], while a “Roma” sample from Lucknow was rich in myrcene (45.6%) [7].

The results from the antimicrobial screening and the cytotoxicity screening of the leaf oil of *C. longa* are summarized in Table 2. The leaf oil demonstrated particularly strong antibacterial activity against the Gram-positive organisms *B. cereus* and *S. aureus* (MIC = 78 μg/mL) and antifungal activity against *A. niger* (MIC = 19.5 μg/mL). The antimicrobial activities observed in *C. longa* leaf oil can be attributed to the major component *ar*-turmerone. Consistent with these results, *ar*-turmerone had previously shown strong antibacterial activity against *Clostridium perfringens* and weak inhibitory activity against *Escherichia coli* [38]. The antimicrobial results in this study showed that Gram-positive bacteria were more sensitive to the leaf oil of *C. longa* (Table 2). This result is consistent with those reported by other workers [39,40,41,42]. According to Alzoreky and Nakahara [42], Gram-negative bacteria are less susceptible to plant extracts due to lipopolysaccharides in their outer membrane. Similarly, turmerone-rich essential oils have shown antifungal activity [43,44], and *ar*-turmerone itself has shown antifungal activity against *Aspergillus flavus* [45].

**Table 2:** Biological activities of *Curcuma longa* leaf essential oil.

**Antimicrobial Activity (MIC, μg/mL)**						
**Sample**	***B. cereus***	***S. aureus***	***E. coli***	***P. aeruginosa***	***C. albicans***	***A. niger***
*C. longa* leaf EO	78	78	312	625	312	19.5
Positive control	1.22 ^a^	0.61 ^a^	2.44 ^a^	1.22^a^	0.61^b^	0.61 ^b^
**Cytotoxic Activity**						
**Sample**	**Hs578T ^c^**			**PC-3 ^d^**		
*C. longa* leaf EO	98.86 ± 0.63			97.94 ± 2.05		
Tingenone	100			100		

^a^ Gentamicin sulfate; ^b^ Amphotericin B; ^c^ % kill at 250 μg/mL; ^d^ % kill at 100 μg/mL; EO = Essential oil.

The observed *in vitro* cytotoxicity against breast tumor (Hs578T) and prostate tumor (PC-3) cells is also consistent with the previously observed cytotoxicity of *ar*-turmerone. Thus, *ar*-turmerone has shown cytotoxic activity to HL-60, K-562, L-1210 [46], HeLa [47], U-937, and RBL-2H3 cells [48]. Furthermore, *ar*-turmerone has been shown to induce apoptosis coupled with DNA fragmentation in several cell lines [48,49,50,51].

## 4. Conclusions

High concentrations of turmerones were identified in the leaf essential oil of *Curcuma longa* from Mbaise, Nigeria. The composition of the leaf oil represents a new chemotype of this plant. The significant antibacterial, antifungal, and cytotoxic activities of the leaf oil may be attributed to the turmerone content. Due to the turmerone-rich essential oil, this chemotype of *C. longa* from southern Nigeria has promising therapeutic potential.
